# The Relationship between Anterior Cruciate Ligament Injury and Osteoarthritis of the Knee

**DOI:** 10.1155/2015/928301

**Published:** 2015-04-19

**Authors:** David Simon, Randy Mascarenhas, Bryan M. Saltzman, Meaghan Rollins, Bernard R. Bach, Peter MacDonald

**Affiliations:** ^1^Pan Am Clinic, Section of Orthopaedic Surgery, University of Manitoba, 75 Poseidon Bay, Winnipeg, MB, Canada R3M 3E4; ^2^Section of Orthopaedic Surgery, Rush University Medical Center, 1611 West Harrison Street, Suite 300, Chicago, IL 60612, USA

## Abstract

Anterior cruciate ligament (ACL) tears are a common injury, particularly in the athletic and youth populations. The known association between ACL injury and subsequent osteoarthritis (OA) of the knee merits a more in-depth understanding of the relationship between the ACL-injured knee and osteoarthritis. ACL injury, especially with concomitant meniscal or other ligamentous pathology, predisposes the knee to an increased risk of osteoarthritis. ACL insufficiency results in deterioration of the normal physiologic knee bending culminating in increased anterior tibial translation and increased internal tibial rotation. This leads to increased mean contact stresses in the posterior medial and lateral compartments under anterior and rotational loading. However, surgical reconstruction of the ACL has not been shown to reduce the risk of future OA development back to baseline and has variability based on operative factors of graft choice, timing of surgery, presence of meniscal and chondral abnormalities, and surgical technique. Known strategies to prevent OA development are applicable to patients with ACL deficiency or after ACL reconstruction and include weight management, avoidance of excessive musculoskeletal loading, and strength training. Reconstruction of the ACL does not necessarily prevent osteoarthritis in many of these patients and may depend on several external variables.

## 1. Introduction

Unlike many tendons and ligaments, a torn anterior cruciate ligament (ACL) rarely heals into its anatomic or physiologic position. It is commonly associated with damage to the menisci, other ligaments, articular cartilage, and subchondral or cancellous bone [1–3]. These associated injuries can occur concurrently with the acute ACL injury, as well as over time in the ACL-deficient knee [1]. Notching can occur in chronic ACL injury from violation and bony loss at the anterolateral femoral condyle due to impaction from the anterolateral and/or posterolateral tibial rim and meniscus in this region [1]. Subchondral sclerosis, meniscal degeneration, and osteochondral defects are also commonly observed in the chronic ACL-deficient knee [1]. Reticular patterns involving medullary edema comprise approximately 70% of such lesions, and geographic patterns of bone bruise have been observed in 66% of the patient population [1–4].

Research demonstrates that adolescents and young adults who sustain an ACL injury are at a substantially increased risk for the development of future osteoarthritis (OA) in the patellofemoral and tibiofemoral joints [1, 3–9]. OA in this situation is defined by objective structural findings including cartilaginous wear or joint-line changes via radiographic imaging or direct visualization. Some studies suggest that as many as 80% of ACL injured knees may demonstrate radiographic evidence of OA at 5 to 15 years after initial injury, especially with concomitant meniscal damage [3, 4, 10–12]. Basic science studies have demonstrated an increase in biomarker concentrations of cartilage turnover after ACL injury, indicating a role in the OA process [13]. Patients with severe radiographic osteoarthritis have poorer health-related quality of life, and as such the clinical impact is significant [14]. Additionally, research has shown that individuals who sustained an ACL injury while playing soccer had a 51% higher chance of developing radiographic changes secondary to OA 12–14 years after injury [15, 16] and that the risk of developing OA increased 100 times in athletes who have sustained a knee injury [17, 18]. In light of these findings, it is not surprising that only one study has shown that knee cartilage can remain preserved 20 years after ACL injury without reconstruction [19].

While injury to knee articular cartilage, menisci, and/or other ligaments is thought to contribute to the development of OA in the ACL-deficient knee, secondary injury due to instability and alterations in the normal biomechanics of the knee is also thought to play a role in the development of OA [9, 20–25]. Therefore, ACL injury has a dramatic impact on the normal kinematics of the knee joint by making it highly susceptible to further injury, chronic instability, and long-term degenerative changes.

Current literature in ACL reconstruction has demonstrated reproducible medium-term favorable clinical results with low complication and graft failure rates, high rates of negative pivot-shift testing, and similar KT-1000 arthrometer measurements between operative and contralateral knees [26]. While surgery aims to reproduce native ACL anatomy, the best attempts at ACL reconstruction continue to fall short of optimally restoring normal kinematics of the knee joint. As a result, secondary lesions and degenerative changes continue to impact the ACL-reconstructed population. A recent meta-analysis of nine long-term studies noted an incidence of 20% (121 of 596) of ACL-injured knees exhibiting moderate or severe radiologic changes (grade III or IV) compared to just 5% (23 of 465) of contralateral uninjured knees. The authors reported that the relative risk of developing minimal osteoarthritic changes following ACL injury was 3.89 regardless of whether or not patients had surgery, while relative risk of developing moderate to severe OA was 3.84 [27]. A recent trial by Barenius et al. [28] reported that at 14-year follow-up from ACL reconstruction an incidence of 57% of OA was significantly greater than the 18% of OA cases in the contralateral knee, with OA most frequently found in the medial compartment. A retrospective case series by Leiter et al. [29] similarly concluded that knees which underwent ACLR had a significantly greater incidence and severity of OA than their non-ACL-injured counterparts. In a cross-sectional study by Roos et al. [30], patients with injury to the ACL showed the first radiologic signs of OA (joint space narrowing) at an average age of about 40 years.

## 2. ACL Structure and Kinematic Function

The primary function of ligamentous structures about the knee is to resist tensile forces in line with their functional axis. Complex knee kinematics include multiple axes of rotation, which are constantly changing under physiologic loads [31]. Therefore, forces across the knee are absorbed and counterbalanced by selective engagement of fiber bundles within the various ligaments in response to the flexion angle and load applied. Consequently, individual ligamentous structures, such as the ACL, may function as primary or secondary stabilizers depending upon the position of the limb in space [32].

The cruciate ligaments are the primary stabilizers of anteroposterior translation of the tibia relative to the femur when the knee is flexed, providing more than 80% of resistance at flexion angles from 30 to 90 degrees [32]. At these flexion angles, structures such as the iliotibial band, collateral ligaments, joint capsule, and menisci provide little to no additional secondary restraint [32]. Anterior tibial translation is the greatest between 20 and 45 degrees of flexion [33]. At flexion angles greater than 90 degrees, both components of the MCL become important anteroposterior stabilizers.

Biomechanical studies have also revealed that sectioning of the ACL results in a significant increase of internal tibial rotation near extension, while additional sectioning of the collateral ligaments produced no further increases, indicating that the ACL is also an important restraint against internal rotation moments during flexion-extension [34, 35]. At increasing flexion angles, anterolateral and posteromedial capsular structures are recruited during internal rotation as the ACL slackens and the posterior cruciate ligament tightens [31].

Between 20 degrees of flexion and full extension, the cruciate ligaments contribute to rotation between the tibia and femur known as the “screw home” mechanism, which is a key element in knee stability for standing upright. During normal gait, the tibia internally rotates during swing phase and external rotation occurs during terminal extension due to the difference in the radius of curvature of the medial and smaller lateral condyle. The net result is tightening of both cruciate ligaments which locks the knee with the tibia in a position of maximal stability with respect to the femur.

### 2.1. The ACL-Deficient Knee

**Figure 1:**
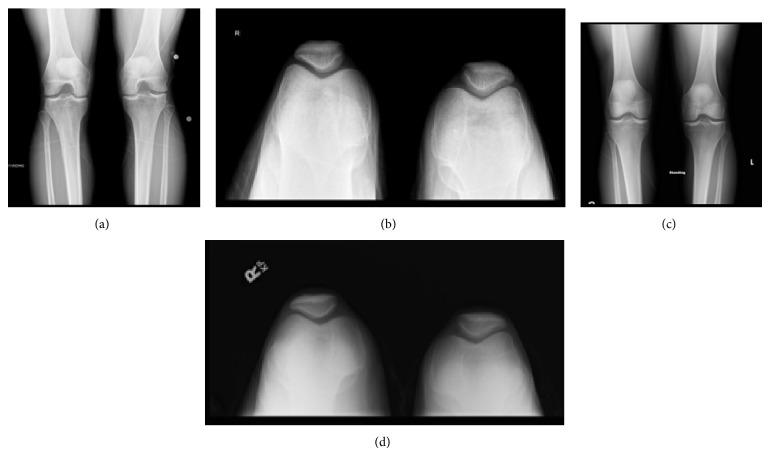
AP and sunrise knee radiographic images of a 29-year-old male patient at (a-b) 8 months and (c-d) 30 months after an acute ACL injury. The images show progression of osteoarthritic changes in a young male with an ACL-deficient knee.

Deficiency of the ACL results in suboptimal kinematics as effective transfer of loads relies on mechanical stability. ACL insufficiency causes deterioration of the physiologic roll-glide mechanism culminating in increased anterior tibial translation as well as increased internal tibial rotation [31]. It results in increased mean contact stress in the medial and lateral compartment posterior sectors under anterior and rotational loads, respectively [36]. With muscular fatigue or poor neuromuscular control, patients experience combined anterior and rotatory instability as a subluxation of the tibiofemoral joint. Ultimately, failure of a primary restraint such as the ACL necessitates recruitment of secondary structures (e.g., menisci) in order to resist external forces and to stabilize joint motion. The higher loads borne by secondary structures may render them more susceptible to degeneration or secondary failure (Figure 1).

Numerous biomechanical studies on the ACL-deficient knee have been performed to better understand the altered kinematics at the knee with this anatomical change. As the knee moves from extension into 70 degrees of knee flexion during wall squatting, the tibia is significantly more internally rotated in an ACL-deficient knee possibly interfering with the “screw-home” mechanism of tibiofemoral kinematics [37]. Three-dimensional tibiofemoral kinematics of the ACL-deficient knee during upright weight-bearing flexion demonstrate posterior subluxation of the lateral femoral condyle at early positions of flexion, with concomitant excess external femoral rotation; the lateral condyle moves slightly posteriorly causing reduced external femoral rotation during flexion from the 15 to 60 degrees arc [38]. During stair ascent and descent, as well as during the entire gait cycle, ACL-deficient knees display a more varus and internally rotated tibial position when compared to ACL-intact knees [39, 40]. Significant reductions in extension were observed during the midstance in ACL-deficient knees [39] but with significantly higher anterior tibial translation and higher flexion angles than the intact contralateral side [41], and significantly decreased flexor and extensor muscle groups about the knee are present [42]. In high-demand activities such as side cutting motions, the ACL-deficient knee increases offset toward less valgus and more external tibial rotation potentially as an adaptation to avoid pivot shift dynamically [43].

There have additionally been multiple biomechanical analyses on the knee after ACLR to assess for restoration of native kinematics about the joint; these have almost uniformly found that abnormalities in kinematics are not eliminated with reconstruction of the ACL. Step length, maximum knee flexion angle during loading response, walking speed, threshold to detect passive motion, and joint position sense are found to be restored after ACLR; however, no significant improvements are observed in maximum angular knee flexion excursion during stance, peak knee flexion moment during walking, peak knee flexion angle, or maximum external tibial rotation angle throughout the gait cycle [44]. Gao et al. found that ACL reconstructed knees were more similar to normal spatiotemporal gait parameters and joint kinematics but still with deficits in comparison to ACL-intact knees [40]. Significant reductions in extension were observed during the swing phase in ACL-deficient knees [39]. The quadriceps remain weak even up to 6 months after ACLR, potentially contributing to altered mechanics about the knee [45].

## 3. Concomitant Bone, Cartilage, and Synovial Pathology with ACL Injury

ACL injury leads to anterior subluxation of the tibia with impaction of the posterior lateral tibial plateau against the anterior aspect of the lateral femoral condyle and can cause significant bony and cartilaginous injury to these regions [46]. Cortical depression fractures seen as differing volumes of bone marrow edema after ACL injury are often present and found to be associated with lower clinical outcome scores at 1 year after ACLR [46]. Rarely, more widespread bone contusions at the inferior patella and anteromedial tibial plateau have been described [47].

Cartilage injury that is associated with ACL injury has been extensively evaluated with advanced imaging studies, including quantitative T(1)p MRI [48]. Potter et al. [49] prospectively evaluated 40 knees with acute, isolated ACL injury and found that all patients sustained chondral injury acutely at the time of ACL tear. With use of morphologic MRI and quantitative T2 mapping, the following adjusted risks of cartilage loss over time were reported: two-time baseline for the lateral compartment and medial femoral condyle and 3-time baseline for the patella at 1 year after injury. Adjusted risks were also fifty-time baseline for the lateral femoral condyle, thirty-time baseline for the patella, and 19-time baseline for the medial femoral condyle at 7 to 11 years after injury. There was additionally an association between the initial size of bone marrow edema pattern and subsequent cartilage degeneration [49]. The cartilage overlying a consequent bone marrow edema-like lesion after ACL injury is reported to have persistent T(1p) MRI signal changes at 1 year after injury despite improvement of the bony changes; these MRI signal changes additionally demonstrate that the superficial layers of cartilage at site of injury have greater matrix damage than the deep layers at the lateral tibia after ACL tear [50]. The MRI T(1)p changes still do not return to baseline over the posterolateral tibial cartilage, in addition to T2 MRI quantitative values in the cartilage over the central medial femoral condyle, even 2 years after ACLR [51]. All of these aforementioned changes may correlate to the eventual development of posttraumatic OA in the knee after ACL injury and ACLR.

It is proposed that synovial biomarkers may provide prognostic indicators of OA in ACL-deficient and reconstructed patients before radiographic damage is evident and may represent a continued inflammatory state of the knee. Synovial fluid biomarkers demonstrate elevated collagen turnover in both deficient and reconstructed patients, and reconstructed patients show continued elevated synovial inflammatory cytokines postoperatively compared to preoperative values [52]. Increased levels of the proinflammatory cytokines Interleukin- (IL-) 6, IL-8, interferon gamma, macrophage inflammatory protein 1B, and monocyte chemotactic protein in the acute phase (as early as 1 day) after ACL injury are hypothesized to play a role in triggering early cartilage catabolism [53, 54]. C-reactive protein (CRP), as a marker of ongoing tissue damage, is additionally elevated significantly at day 3 after an ACL injury before returning thereafter to preinjury normal values [55]. Elevated serum levels of a chondroitin sulfate epitope WF6 are additionally found in patients after ACL injury and may be helpful in the future as early assays for detection of posttraumatic OA development [56].

## 4. Knee-Related Risk Factors

### 4.1. Meniscectomy

**Figure 2:**
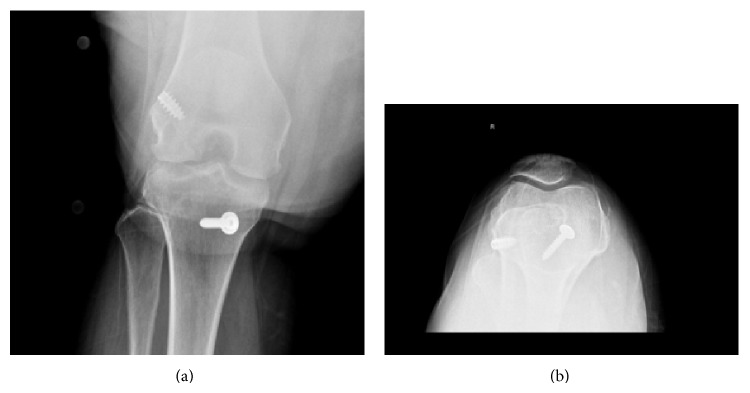
(a) Anteroposterior (AP) and (b) sunrise view of the right knee in a 39-year-old female patient who underwent ACL reconstruction with medial meniscectomy at the age of 33 after sustaining an ACL tear with concomitant medial meniscus and MCL tears. These radiographs demonstrate joint space narrowing, particularly in the medial and patellofemoral compartments, consistent with early osteoarthritic changes.

**Figure 3:**
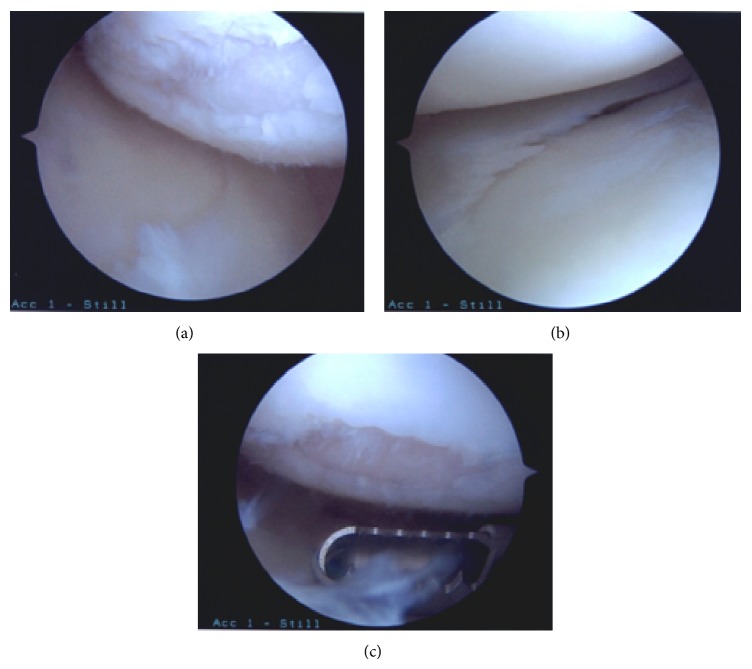
Arthroscopic images depicting arthritic changes in the same 39-year-old female patient described in Figure 1, who underwent ACL reconstruction with medial meniscectomy at the age of 33 after sustaining an ACL tear with concomitant medial meniscus and MCL tears.

Approximately 50% of ACL tears are believed to be accompanied by meniscal injury at the time of the acute injury, while in the chronic ACL-deficient knee, meniscal tears have been observed in as high as 80% of the patient population [3, 9]. Meniscectomy might be the most important risk factor for developing knee osteoarthritis after an ACL injury (Figures 2 and 3). A review of risk factors responsible for the development of knee OA after surgical management of meniscal tears highlighted a significantly higher outcome score regarding osteoarthritis when partial meniscectomy was performed compared to subtotal and total meniscectomy [57]. The authors thus concluded that the amount of meniscus resected was the most important surgical predictive factor for the development of OA. In evaluating the risk factors predictive of tibiofemoral OA after ACLR, the strongest discriminator was meniscectomy per the cohort study by Keays et al. [58]; this was also the strongest predictor of patellofemoral OA. In their systematic review of the literature, Øiestad et al. [59] detailed that the most frequently reported risk factor for the development of knee OA was meniscal injury (defined as either meniscectomy, meniscal tear, or meniscal surgery) in the 7 prospective and 24 retrospective studies included. A nested cohort analysis within the MOON (Multicenter Orthopaedic Outcomes Network) database determined that ACL reconstructed knees with meniscectomy had more narrow minimum joint space width compared to their contralateral normal knees [60].

### 4.2. Graft Choice

A systematic review of autograft choice comparing hamstring and patellar tendon autografts identified no difference between grafts in clinical assessment or patient-reported outcomes [61]. ACL reconstruction provides good short and intermediate-term results, regardless of graft used [62–64], but degenerative changes in knee cartilage can become apparent with time after surgery [65] and there is potential for increased incidence of OA in the patellar tendon group [61, 66]. A prospective comparison study of hamstring and patellar tendon autograft has additionally demonstrated significantly higher rates of radiologically detectable patellofemoral OA (grade A: 46% in patellar tendon and 69% in hamstring tendon use) [67]. Barenius et al. [28] did not report any significant difference in OA of the medial compartment with use of BPTB or quadrupled semitendinosus tendon graft at 14 years after ACLR, although the data trended toward higher OA after BPTB as well (65% versus 49%). This association with less OA may be a result of the lessened relative alteration in native knee joint mechanics inherent to hamstring graft harvest [7], but this relationship is controversial and has yet to be definitively proven in the literature. In evaluating the risk factors predictive of tibiofemoral OA after ACLR, patellar tendon grafts were the second strongest discriminator per the cohort study by Keays et al. [58].

### 4.3. Conservative versus Surgical Treatment

ACL injury alone is a well-known risk factor for the development of knee OA with or without reconstruction [68]. Retrospectively, the rates of radiographic OA and limitations in activities of daily living are the highest in nonreconstructed patients with concomitant knee injuries. The authors also found that ACL reconstruction did not prevent the development of OA but did lead to a lower prevalence of its onset in some studies [69]. Other studies, by contrast, have found more radiographic evidence of OA changes in surgically repaired ACL cohorts when compared to those with chronic ACL deficiency treated nonoperatively [70]. Any damage sustained after ACL injury has important clinical implications when ACL reconstruction is being considered. A systematic review of the literature illustrated that surgical reconstruction of the ACL is superior to conservative treatment [11, 71] because it offers the best approach for reestablishing normal joint kinematics and structural integrity and thus minimizing the likelihood of the affected knee suffering further joint injury or deterioration [71]. While multiple factors confound the issue including concomitant meniscal injury, surgical technique, and patient activity levels [7], the meta-analysis by Aljuied et al. [27] reported a significantly higher relative risk (4.98) of developing any grade of OA after nonoperative treatment of an ACL-injured knee than those treated with reconstruction (3.62). Additionally, revision reconstruction patients have been shown to have more signs of OA and worsened quality of life than their primary counterparts [72]. A recent systematic review of the literature by Chalmers et al. [73], however, did not find any significant differences in radiographically evident OA in a cohort of 1484 total patients who had undergone ACLR versus 685 patients who had been treated nonoperatively.

### 4.4. Timing of Surgery

Research has also shown worse surgical outcome for delayed ACL reconstruction (ACLR) compared to subacute reconstruction. Sernert et al. [74] found an increase in meniscal tears combined with poorer outcome in patients who underwent delayed ACL reconstruction compared to those who were reconstructed subacutely. Results from the Markov decision model analysis by Mather et al. [75] using outcome probabilities and effectiveness derived from the KANON (knee anterior cruciate ligament, nonsurgical versus surgical treatment) and MOON databases found an incremental gain of 0.28 QALYs (quality-adjusted life years) at a lower overall cost to society of $1572 with early ACLR than with rehabilitation plus optional delayed ACLR. In the pediatric patient, meta-analysis has additionally revealed multiple trends favoring early surgical stabilization over nonoperative or delayed ACLR as the latter experienced more instability or pathological laxity and an inability to return to previous levels of activity [76]. In the prospective randomized clinical trial comparing early versus delayed ACLR by Bottoni et al. [77], the range of motion, operative time, KT-1000 arthrometer differences, and subjective knee evaluations were not significantly different between the two cohorts. Although these analyses do not report on osteoarthritis changes when comparing the timing of ACLR, a prospective analysis by Jomha et al. [78] of 72 patients at 7 years after arthroscopic BPTB ACLR determined that early reconstruction of ACL-deficient knees demonstrated the lowest incidence of degenerative changes on radiographic follow-up. By contrast, Harris et al. [79] concluded that at 5 years after ACLR, early ACLR did not provide superior results and had a higher proportion of development of tibiofemoral radiographic osteoarthritis (16% versus 7%) than did delayed ACLR in a cohort of 121 moderately active adults.

### 4.5. Double versus Single Bundle Reconstruction

Trials comparing osteoarthritis after double bundle versus single bundle ACL reconstruction are limited but increasing in number given the superior rotational control after double bundle reconstruction which may better restore knee rotational kinematics during functional activity [7]. However, Ventura et al. [80] retrospectively compared 36 patients who underwent single bundle reconstruction to 14 patients who underwent double bundle reconstruction and reported no difference in the rate of radiological osteoarthritic changes at a mean of 4.4 years postoperatively. The results from Suomalainen et al. [81] were similar in their prospective study of 90 patients at 5-year follow-up. Likewise, Song et al. [82] did not find a difference between techniques in preventing OA in their prospective randomized controlled trial, with 9.6% of patients in the double bundle cohort and 10% in the single bundle cohort exhibiting findings of more advanced OA at final follow-up. Additionally, no significant differences were observed in Knee injury and Osteoarthritis Outcome Scores (KOOS) between techniques in prospective randomized studies by Zhang et al. [83], Ahldén et al. [84], and Aglietti et al. [85].

## 5. Demographic Risk Factors

### 5.1. Residual Laxity/Muscle Weakness

Muscles around the knee act to facilitate knee mobility and stability, as well as aid in force transfer across the knee joint. Muscle weakness is associated with the development of OA [86, 87] and may be one of the earliest and most commonly observed findings in patients with OA [86]. In evaluating the risk factors predictive of tibiofemoral OA after ACLR, weak quadriceps and low quadriceps-to-hamstring strength ratios were very close discriminators per the cohort study by Keays et al. [58]. Early ACL injury-prevention protocols focused on enhancing the protection offered to the knee joint by the hamstrings, but research by Simonsen et al. [88] has shown that they may be ineffective in protecting knee ligaments due to delayed neuromuscular response. Nevertheless, a prospective cohort study by Tourville et al. [89] demonstrated that patients who had undergone ACL reconstruction and had documented quadriceps muscle weakness after surgery had significantly narrowed radiographic tibiofemoral joint space at four-year follow-up, perhaps characterizing the onset of posttraumatic osteoarthritis before the clinical manifestation of the disease. Patients with previous ACL injuries may benefit from exercise interventions to prevent or delay the progression of OA [90], including quadriceps strengthening. Muscle function is rarely fully restored in ACL-deficient patients regardless of whether surgical reconstruction has taken place and this resultant weakness is considered a potential contributor to OA development. Neuromuscular knee rehabilitation and subsequent strengthening and proprioception awareness have been related to a low prevalence of radiographic knee OA in patients with ACL injury treated without reconstruction [91].

### 5.2. Age

With regards to the ACL, age greater than 50 significantly increases (hazard ratio of 37.28 compared to age less than 50 years) the risk of osteoarthritic changes requiring knee arthroplasty at fifteen years status after ACL reconstruction [92]. Older patients at the time of ACLR have been demonstrated to have greater degrees of patellofemoral OA at follow-up 12 years after ACLR [93]. However, individual studies have demonstrated that the level of OA does not statistically increase at more than 32 months after ALCR in patients over the age of 50 years old [80]. The aforementioned nested MOON cohort study by Jones et al. [60] additionally found a significant association between older age and narrower radiographic joint space width from 2 to 3 years after ACLR. In evaluating the risk factors predictive of patellofemoral OA after ACLR, older age at surgery was a defined discriminator per the cohort study by Keays et al. [58]. In the animal model, cartilage degradation has been shown to be significantly higher in middle-aged rats rather than young rats after ACL transection [94].

### 5.3. Gender

It has been reported that female gender is an important risk factor to the occurrence of ACL injury [95]. Additionally, female sex has been reported to have an association with radiographic knee OA after ACLR [96]. Recent data has also demonstrated that female gender has a marked effect (hazard ratio of 1.58 compared to male gender) on the risk of post-ACL reconstruction patients requiring knee arthroplasty after fifteen years [92].

### 5.4. Knee Alignment

Interestingly, varus alignment of the uninjured knee has been demonstrated to have an association with OA in the ACL-injured knee at 15 years after injury according to data from Swärd et al. [97]. Development of degenerative changes after ACL injury was associated with varus deformity knees in the cohort evaluation by McDaniel Jr. and Dameron Jr. [98]. In a comparison of patients undergoing revision and primary ACLR, Won et al. [99] demonstrated that patients undergoing revision ACLR had more frequent varus malalignment and this was associated with a greater tendency for higher-grade radiographic OA at the medial tibiofemoral joint. Sagittal displacement of the tibia was evaluated by Egund and Friden [100], who reported in a cohort study of 29 patients that 5 of the 11 patients with sagittal displacement of between 10 and 19 mm had developed early OA at 10 years after surgery despite age ranges from 23 to 28 years old. The occurrence of malalignment as a consequence of ACL injury is seen in the report by Dejour et al. [101] which demonstrated that the development of varus deformity, characterizing progressive OA, has its origination in the wear of the posteromedial tibial plateau due to ACL laxity.

## 6. Prevention

The prevention of knee OA in individuals with ACL injury who undergo nonsurgical or operative reconstruction treatment options is a topic of current study. Efforts are limited at this time primarily to controlled laboratory studies. Shen et al. [102] recently demonstrated that after 18 months, a knitted silk-collagen sponge scaffold used in a rabbit ACL injury model had enhanced expression of ligament genes and better microstructural morphology. This effectively protected articular cartilage and preserved joint space over the postoperative time period, thus suggesting its clinical use as a functional bioscaffold for preventing OA in the setting of ACL reconstruction. Murray and Fleming [103] performed a controlled laboratory study on Yucatan minipigs which demonstrated that bioenhanced (bioactive scaffold used to stimulate healing) ACL repair may provide a new, less invasive treatment option that reduces macroscopic cartilage damage and thus progression of OA postoperatively. Finally, Jean et al. [104] demonstrated on Wistar rats that intraarticular injection of hyaluronic acid limited articular cartilage and synovium damage, reduced excitatory amino acid neurotransmitter levels, and ultimately decreased OA development in the ACL-transected knee suggesting a potential link to its clinical utility for prolonging or eliminating the early development of OA in ACL-deficient individuals.

## 7. Conclusion

ACL injury, especially with concomitant meniscal or other ligamentous pathology, predisposes to an increased risk of osteoarthritic changes at the knee joint. Deficiency of the ACL results in suboptimal kinematics since effective load transfer relies on mechanical stability. Evidence has demonstrated that ACL reconstruction does not necessarily prevent this increased risk for cartilage degradation and depends on such factors as graft choice, timing of surgery, and surgical technique. General prevention of OA changes with weight management, avoidance of excessive loading, and strength training of the surrounding muscles are especially relevant to this patient population.
